# Supplementary material for: Unraveling the drivers of leptospirosis risk in Thailand using machine learning
Source: PLoS Negl Trop Dis. 2025 Oct 14;19(10):e0013618. doi: 10.1371/journal.pntd.0013618 (PMC12539691; doi:10.1371/journal.pntd.0013618)
Supplement: S1 Text — (PDF) [file pntd.0013618.s001.pdf]

Supplementary information:

Unraveling the drivers of leptospirosis risk in Thailand using machine learning

Pikkanet Suttirat[1,2], Sudarat Chadsuthi[3], Charin Modchang[1,2,4]*, Joacim Rocklöv[5,6,7]*

[1]Biophysics Group, Department of Physics, Faculty of Science, Mahidol University, Bangkok, Thailand

[2]Center for Disease Modeling, Faculty of Science, Mahidol University, Bangkok, Thailand

[3]Department of Physics, Faculty of Science, Naresuan University, Phitsanulok, Thailand

[4]Centre of Excellence in Mathematics, MHESI, Bangkok, Thailand

[5]Department of Epidemiology and Global Health, Umeå University, Umeå, Sweden

[6]Heidelberg Institute of Global Health, Heidelberg University, Heidelberg, Germany

[7]Interdisciplinary Center of Scientific Computing, Heidelberg University, Heidelberg, Germany

*__Corresponding Authors:__ Charin Modchang and Joacim Rocklöv

Email: charin.mod@mahidol.edu (CM), joacim.rocklov@umu.se (JR)

S1 Text: XGBoost hyperparameters

In our work, we tuned several key hyperparameters to optimize the XGBoost model's performance and prevent overfitting. The *n_estimators* parameter determines the number of trees grown for the classification task, while the *learning_rate* controls the rate at which the model learns and corrects errors for each new tree. The *max_depth* parameter sets the maximum depth of each tree, with deeper trees more likely to overfit the data and shallower trees (stumps) potentially underfitting. *Subsample* and *colsample_bytree* introduce randomness by subsampling the training data and feature columns, respectively, for each tree, reducing the likelihood of overfitting. The *min_child_weight* parameter sets the minimum number of samples required in each leaf node, further preventing overfitting. *Gamma* serves as a regularization parameter in the loss function, while *alpha* and *lambda* control the L1 and L2 regularization terms on the weights, respectively. Careful tuning of these hyperparameters is crucial to ensure optimal model performance and generalization to unseen data [1].

References

1. Chen T, Guestrin C. XGBoost: A Scalable Tree Boosting System. Proceedings of the 22nd ACM SIGKDD International Conference on Knowledge Discovery and Data Mining; San Francisco, California, USA: Association for Computing Machinery; 2016. p. 785–94.
