# Supplementary figures and images for: Unraveling the drivers of leptospirosis risk in Thailand using machine learning
Source: PLoS Negl Trop Dis. 2025 Oct 14;19(10):e0013618. doi: 10.1371/journal.pntd.0013618 (PMC12539691; doi:10.1371/journal.pntd.0013618)

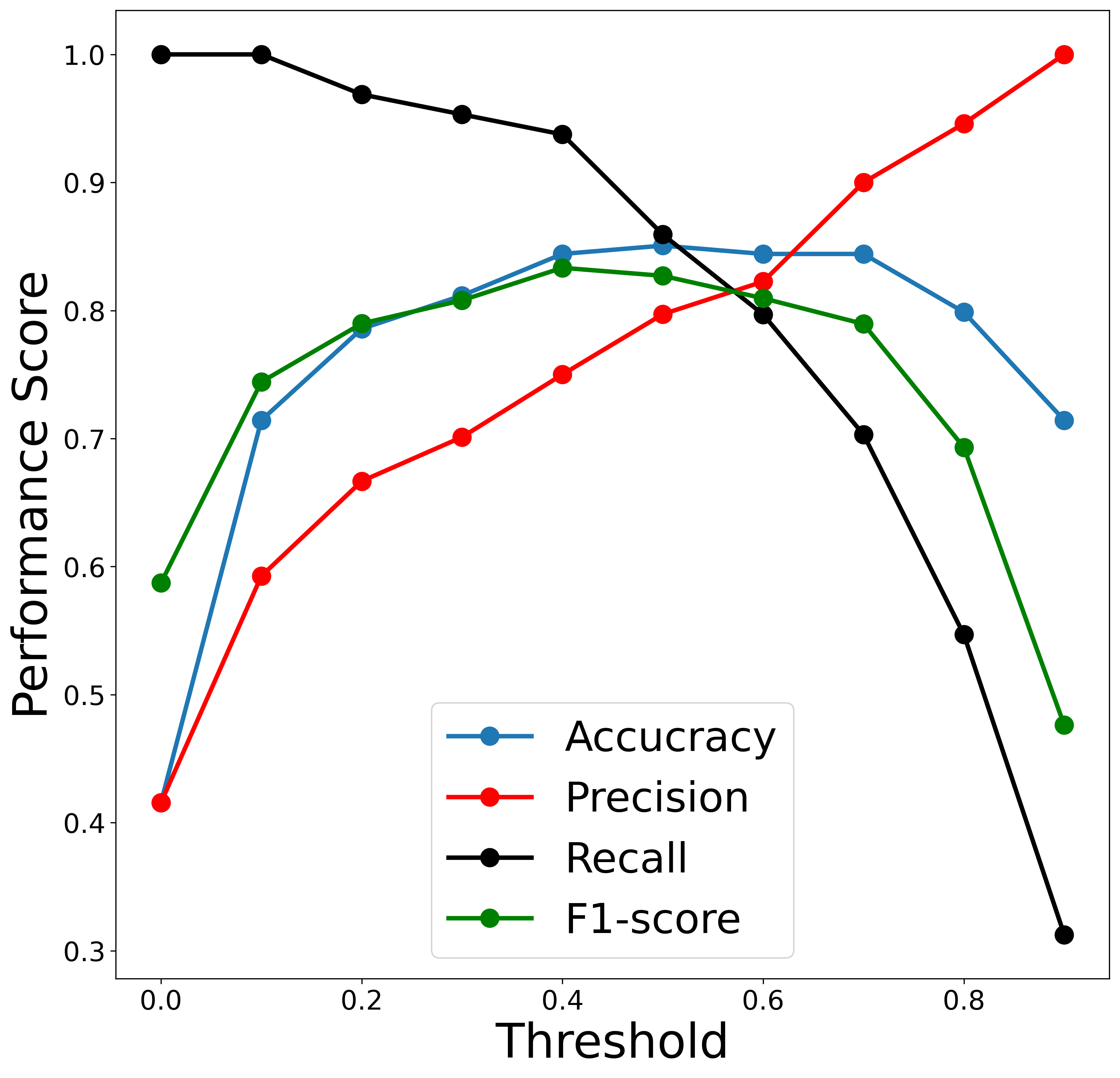

Supplement: S1 Fig — Performance metrics (accuracy, precision, sensitivity, and F1-score) are evaluated across different classification thresholds, where the threshold determines the cutoff between predicted low-risk (0) and high-risk (1) provinces. For each threshold value, provinces are classified as high-risk when their predicted probability exceeds the threshold. (TIF) [file pntd.0013618.s002.tif]

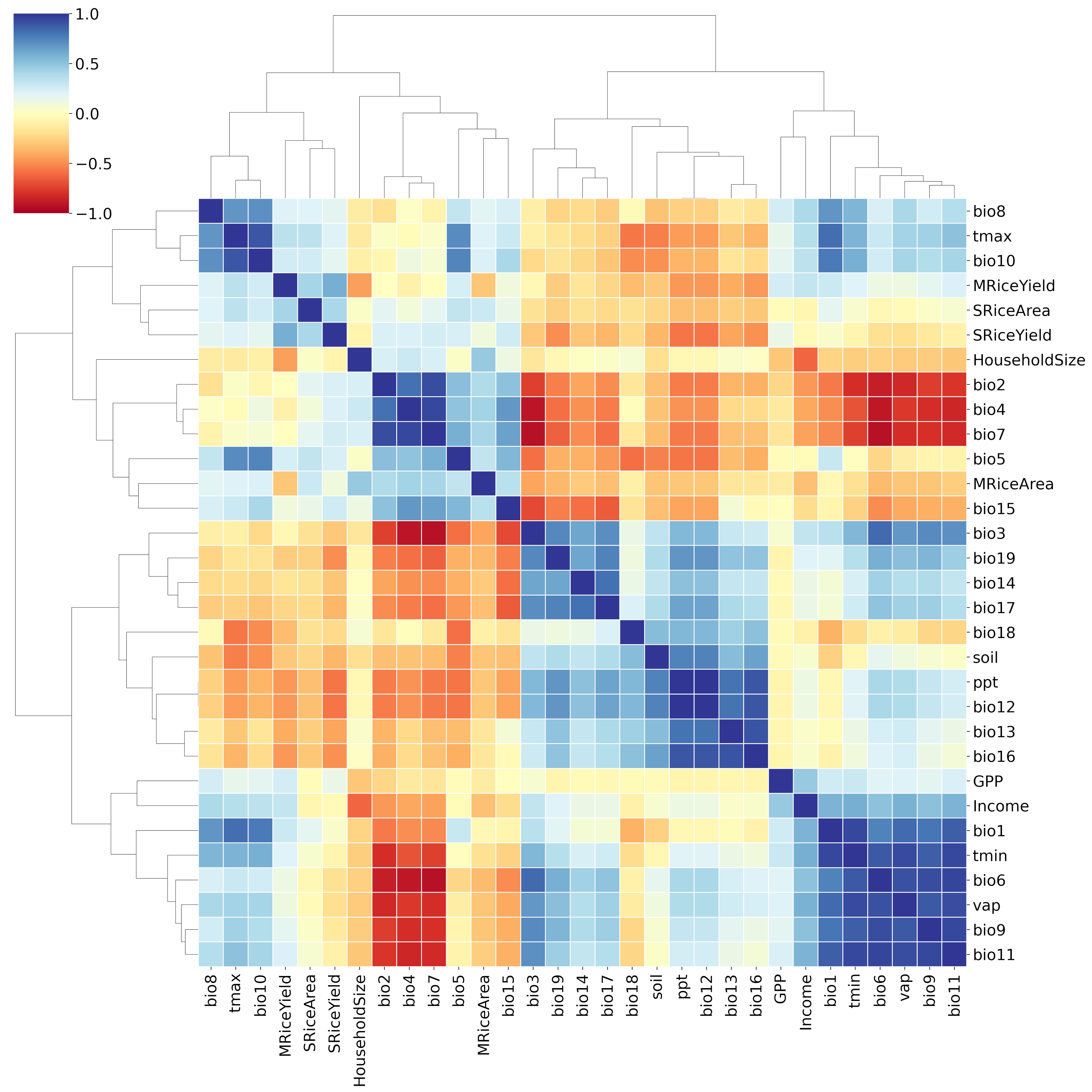

Supplement: S2 Fig — The heatmap displays Pearson correlation coefficients between all model features, visualized using seaborn’s clustermap function. Correlation strength and direction are indicated by color intensity (red = positive, blue = negative). Features are hierarchically clustered to reveal groups of highly correlated variables, highlighting potential multicollinearity in the dataset. (TIF) [file pntd.0013618.s003.tif]

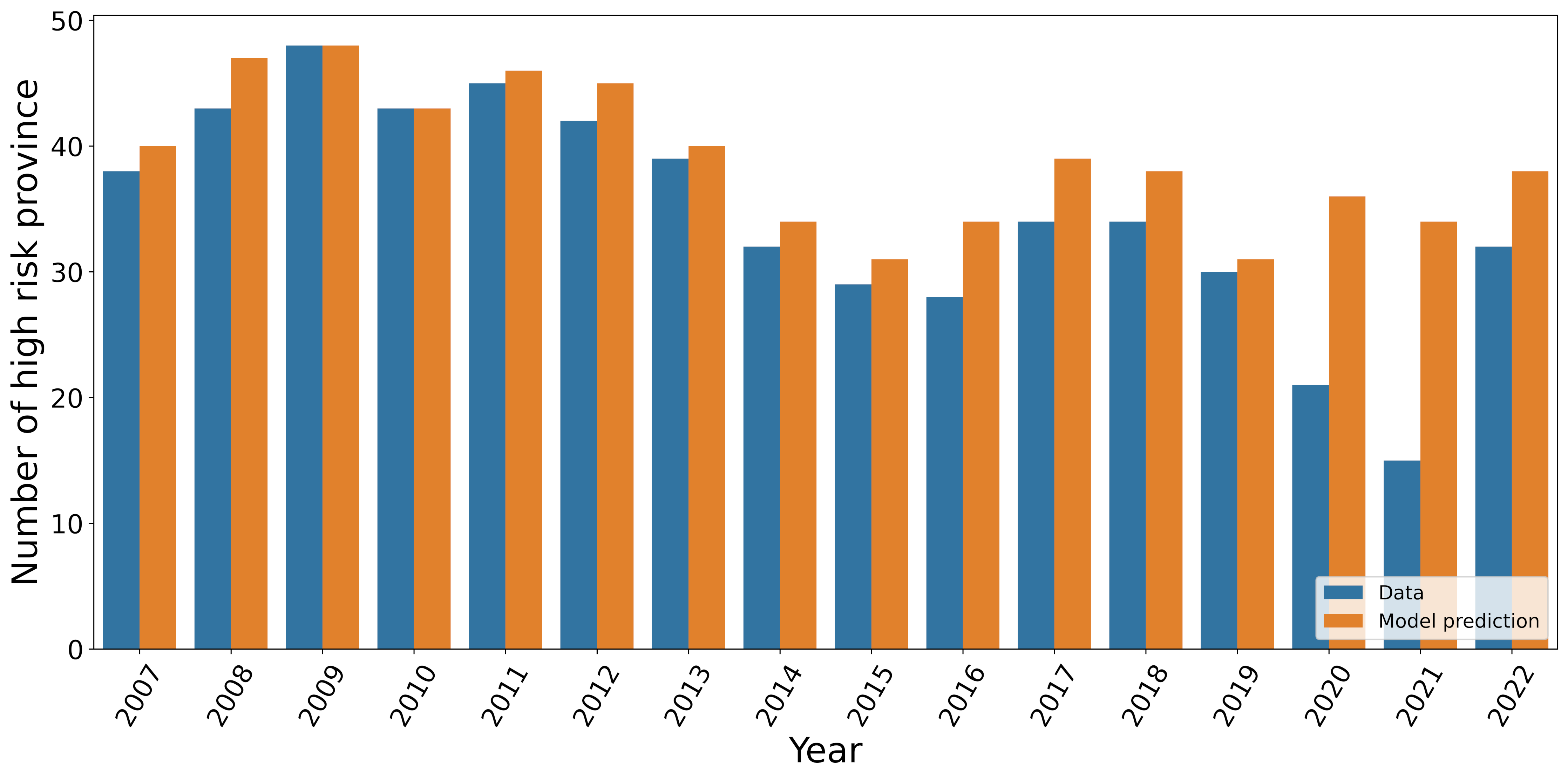

Supplement: S3 Fig — Comparison of model-predicted versus observed counts of high-risk provinces during pre-pandemic (2007–2019), pandemic (2020–2021), and post-pandemic (2022) periods. Using the model trained on 2007–2017 data, predictions reveal substantial changes in the number of high-risk provinces, particularly during the pandemic period. Using a classification threshold of 0.5, the model predicted 36 and 34 high-risk provinces for 2020 and 2021, respectively, while actual data showed only 21 and 15 high-risk provinces. This overestimation suggests a substantial reduction in leptospirosis risk during the pandemic period, independent of climatic conditions. This visualization highlights the pandemic’s potential impact on leptospirosis risk patterns and/or surveillance capabilities across Thailand’s provinces. (TIF) [file pntd.0013618.s004.tif]

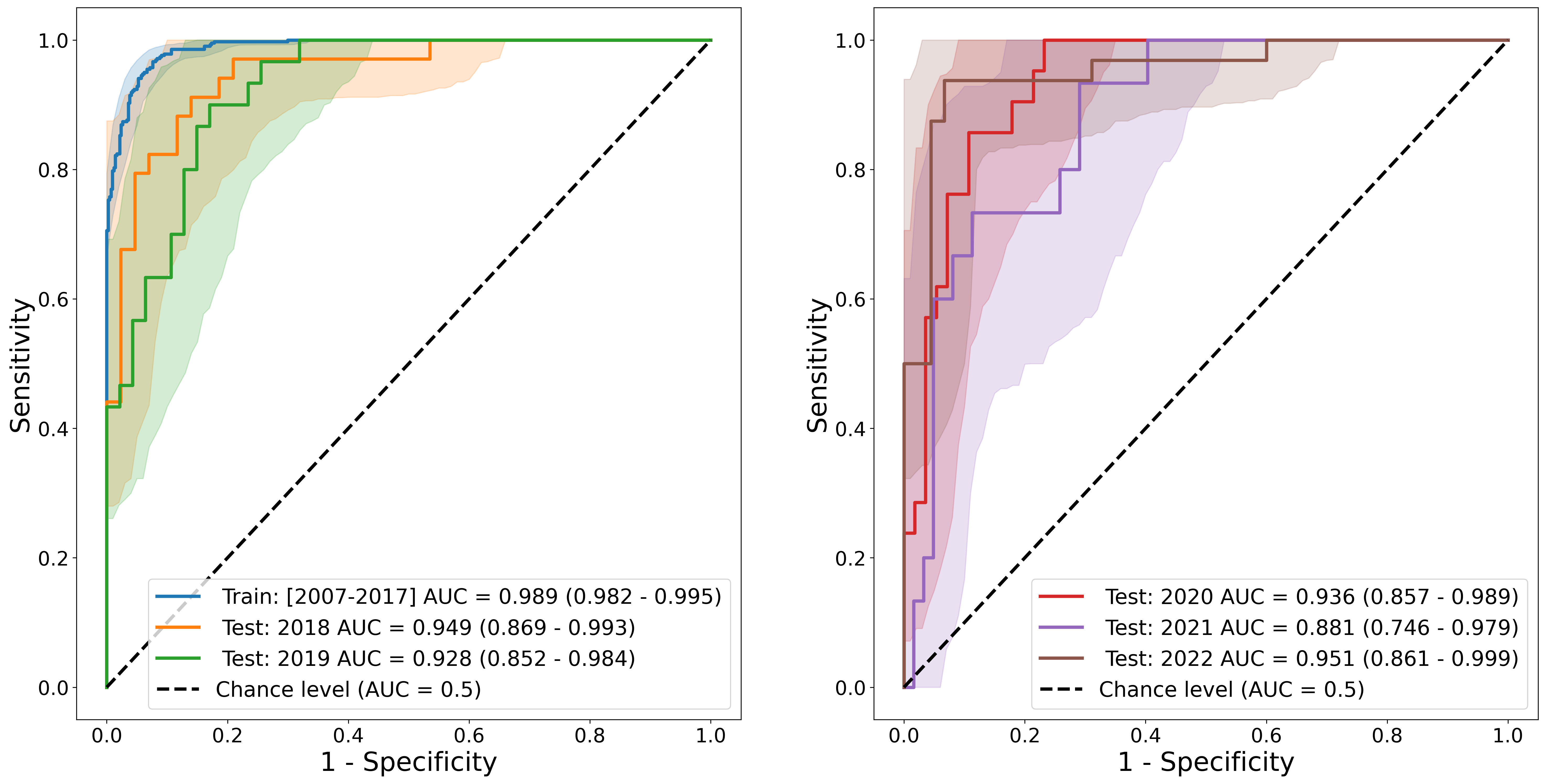

Supplement: S4 Fig — The diagonal reference line represents random classification (AUC = 0.5), while perfect classification corresponds to AUC = 1.0. Shaded areas indicate 95% confidence intervals based on 1,000 bootstrap iterations. 95% CIs of AUC are given in parentheses. (TIF) [file pntd.0013618.s005.tif]

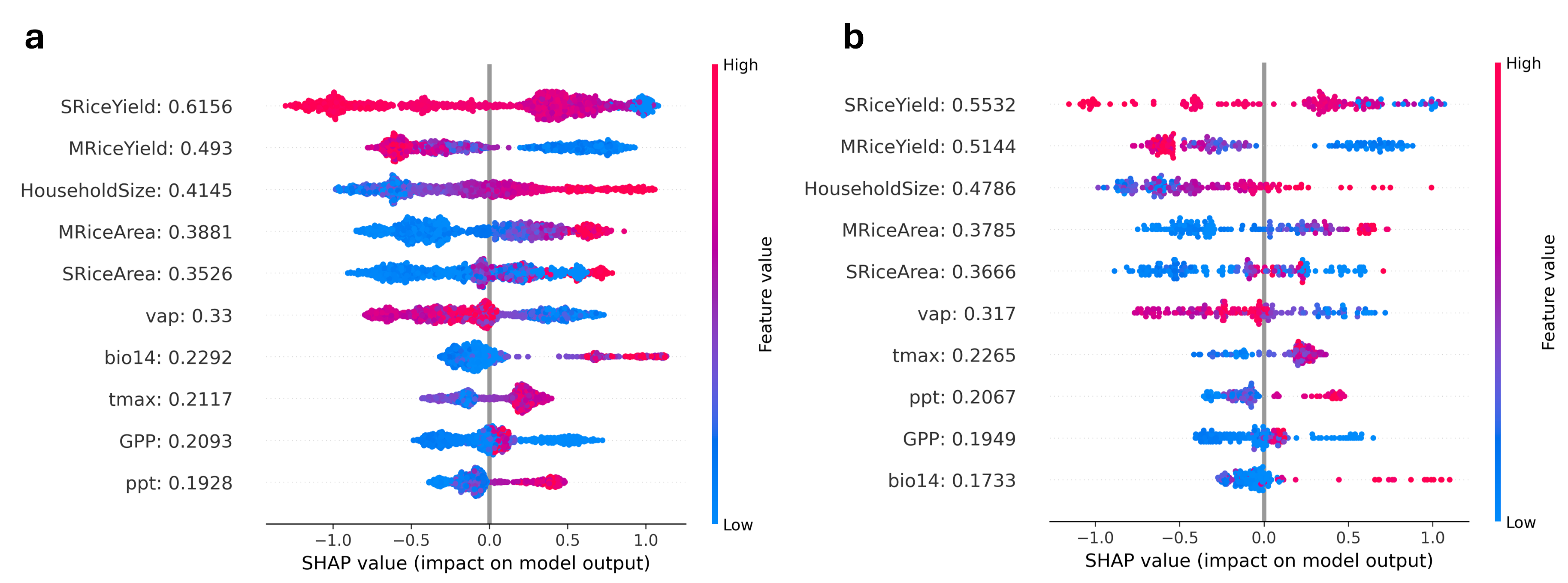

Supplement: S5 Fig — (a) Full dataset covering 2007–2022. (b) during COVID-19 pandemic data only (2020–2021). (TIF) [file pntd.0013618.s006.tif]
