# Supplementary material for: Unraveling the drivers of leptospirosis risk in Thailand using machine learning
Source: PLoS Negl Trop Dis. 2025 Oct 14;19(10):e0013618. doi: 10.1371/journal.pntd.0013618 (PMC12539691; doi:10.1371/journal.pntd.0013618)
Supplement: S1 Table — For each metric, ranking score calculated by summation of assigned rank from best (score = 4) to worst (score = 1) for each metric. The average performance of each model was obtained from average over 10 random seeds. All models were trained on the same training set (2007–2015) to ensure fair comparison. Hyperparameters were optimized using 5-fold cross-validation. (PDF) [file pntd.0013618.s007.pdf]

S1 Table. Performance comparison of four machine learning models (XGBoost, logistic regression, support vector machine, and random forest) using six metrics on training set, pre-COVID-19 (2018-2019) test set, and post-COVID-19 (2022) test set. For each metric, ranking score calculated by summation of assigned rank from best (score = 4) to worst (score = 1) for each metric. The average performance of each model was obtained from average over 10 random seeds. All models were trained on the same training set (2007-2015) to ensure fair comparison. Hyperparameters were optimized using 5-fold cross-validation.

**Classification Model**	**Sets**	**Average performance metrics**						**Ranking Score**
		**AUC**	**Log loss**	**F1**	**Precision**	**Recall**	**Accuracy**	
Logistic regression	Training	0.898	0.405	0.856	0.826	0.889	0.844	6
Support vector machine	Training	0.949	0.299	0.891	0.875	0.908	0.884	12
Random forest	Training	**1.000**	**0.092**	**0.999**	**0.999**	**1.000**	**0.999**	**24**
XGBoost	Training	0.994	0.169	0.961	0.943	0.98	0.959	18
Logistic regression	Pre-COVID-19	0.899	0.406	0.781	0.781	0.781	0.818	8
Support vector machine	Pre-COVID-19	0.933	0.351	**0.845**	0.764	**0.945**	0.856	18
Random forest	Pre-COVID-19	0.915	0.382	0.832	0.776	0.895	0.849	13
XGBoost	Pre-COVID-19	**0.935**	**0.330**	0.835	**0.798**	0.877	**0.856**	**21**
Logistic regression	post-COVID-19	0.894	0.477	0.775	0.646	**0.969**	0.766	9
Support vector machine	post-COVID-19	0.920	0.371	0.796	0.708	0.909	0.806	12
Random forest	post-COVID-19	0.951	0.334	0.868	**0.847**	0.891	**0.887**	18
XGBoost	post-COVID-19	**0.951**	**0.305**	**0.871**	0.814	0.938	0.884	**21**
