# Supplementary material for: Unraveling the drivers of leptospirosis risk in Thailand using machine learning
Source: PLoS Negl Trop Dis. 2025 Oct 14;19(10):e0013618. doi: 10.1371/journal.pntd.0013618 (PMC12539691; doi:10.1371/journal.pntd.0013618)
Supplement: Attachment — Submitted filename: Response Letter_12Sep2025.pdf [file pntd.0013618.s009.pdf]

Response to the reviewers' comments

We would like to submit a revised version of our manuscript entitled "Unraveling the drivers of leptospirosis risk in Thailand using machine learning." We are grateful for the comments and suggestions from the reviewers. We have carefully addressed all comments and made substantial revisions to improve the quality of our manuscript. Please find below our point-by-point response to the comments.

Editorial comments

1. Figure 1B and 1C. Please (a) provide a direct link to the base layer of the map (i.e., the country or region border shape) and ensure this is also included in the figure legend; and (b) provide a link to the terms of use / license information for the base layer image or shapefile. We cannot publish proprietary or copyrighted maps (e.g. Google Maps, Mapquest) and the terms of use for your map base layer must be compatible with our CC BY 4.0 license. Note: if you created the map in a software program like R or ArcGIS, please locate and indicate the source of the basemap shapefile onto which data has been plotted. **Response:** We apologize for the omission of this information. The maps were generated using the plotnine package in Python, utilizing a shapefile obtained from The Humanitarian Data Exchange (HDX) (https://data.humdata.org/dataset/cod-ab-tha). This shapefile is provided under the Creative Commons Attribution for Intergovernmental Organisations (CC BY-IGO) license. To properly acknowledge this source, we have added the following statement to the Figure 1 caption:

- The map plots were created using the plotnine package in Python using a shapefile obtained from The Humanitarian Data Exchange (HDX) (https://data.humdata.org/dataset/cod-ab-tha).

Reviewers' Comments:

Reviewer #1

Comment1: There is a small typo in the code on line 49 (the name of the meta data file).

Response: We apologize for the error and have thoroughly rechecked the code to correct the typo. During this review, we identified an additional issue in Figure 3a (line 161): the plot was incorrectly using data from 2007-2015 (X_train and y_train) rather than the intended 2007-2017 period (X_train_val and y_train_val). We have corrected this error, which resulted in a minor change in AUC from 0.995 to 0.989 (Figure R1 below). Both Figure 3a and all corresponding training AUC values throughout the manuscript have been updated accordingly. Additionally, Figure 3a now includes confidence intervals for the AUC values, as suggested in Comment 3 by Reviewer #2.

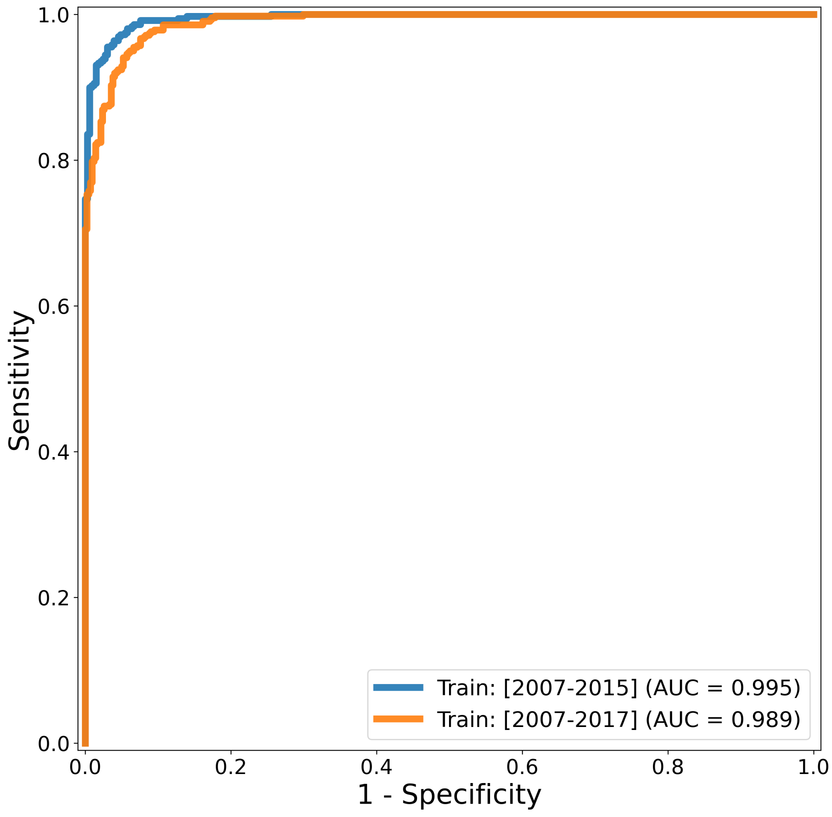

Figure R1: Comparison of XGBoost model performance metrics between 2-time spans: 2007 – 2015 (original version) and 2007 – 2017 (revised version).

Comment 2: The paper is nicely formatted and clear on the goals and the overall results. However, it would be nice to see an extra experiment done where a more basic model is fit to justify the usage of a more complex model such as xgboost. This should be relatively straight forward experiment to run.

Response: We thank the reviewer for this valuable suggestion. We conducted numerical experiments comparing XGBoost with three simpler machine learning models: support vector machine (SVM), logistic regression (LR), and random forest (RF). All models were trained on the same dataset to ensure fair comparison. Features were standardized prior to application in SVM and LR, as these models are sensitive to feature scaling, while tree-based models (random forest and XGBoost) are inherently unaffected by standardization. Hyperparameters for each model were optimized using Bayesian search.

We then computed and compared six performance metrics: AUC, log loss, F1 score, precision, recall, and accuracy. For each metric, models were ranked from best (score = 4) to worst (score = 1), with the overall ranking score calculated as the sum of assigned ranks across all metrics. Our results demonstrate that XGBoost consistently outperformed the other algorithms during the testing periods without exhibiting the overfitting behavior observed in the random forest model, which achieved perfect training performance but lower test performance.

To highlight these findings, we have added Table S1 to the supplementary material. Additionally, the following text has been incorporated into the Discussion section (Lines: 439-442):

- In fact, our preliminary analysis comparing XGBoost, logistic regression, support vector machine, and random forest confirmed that XGBoost outperformed all other algorithms during both pre-COVID-19 and post-COVID-19 testing periods (Table S1).

Table S1. Performance comparison of four machine learning models (XGBoost, logistic regression, support vector machine, and random forest) evaluated across six metrics on three datasets: the training set, pre-COVID-19 test set (2018–2019), and post-COVID-19 test set (2022). Performance metrics include AUC, log loss, F1-score, precision, recall, and accuracy. For each metric, models were ranked from best (score = 4) to worst (score = 1), with the ranking score representing the sum of ranks across all metrics. Model performance values represent averages across 10 random seeds to ensure stability. All models were trained on an identical training dataset (2007–2015) to ensure fair comparison, with hyperparameters optimized through 5-fold cross-validation.

Classification Model	Sets	Average performance metrics						Ranking Score
		AUC	Log loss	F1	Precision	Recall	Accuracy	
Logistic regression	Training	0.898	5.606	0.856	0.826	0.889	0.844	6
Support vector machine	Training	0.949	4.170	0.891	0.875	0.908	0.884	12
Random forest	Training	**1.000**	**0.026**	**0.999**	**0.999**	**1.000**	**0.999**	**24**
XGBoost	Training	0.994	1.483	0.961	0.943	0.98	0.959	18
Logistic regression	Pre-COVID-19	0.899	6.553	0.781	0.781	0.781	0.818	8
Support vector machine	Pre-COVID-19	0.933	5.196	**0.845**	0.764	**0.945**	0.856	18
Random forest	Pre-COVID-19	0.915	5.430	0.832	0.776	0.895	0.849	13
XGBoost	Pre-COVID-19	**0.935**	**5.172**	0.835	**0.798**	0.877	**0.856**	**21**
Logistic regression	post-COVID-19	0.894	8.426	0.775	0.646	**0.969**	0.766	9
Support vector machine	post-COVID-19	0.920	6.975	0.796	0.708	0.909	0.806	12
Random forest	post-COVID-19	0.951	**4.072**	0.868	**0.847**	0.891	**0.887**	19
XGBoost	post-COVID-19	**0.951**	4.166	**0.871**	0.814	0.938	0.884	**20**

Comment 3: Another point which I wouldn't mind addressed either as future work/limitation or another experiment is how well the model can fit the incidence rate. The usefulness of this is that it would allow a user/stakeholder to decide what high to low risk means.

Response: Thank you for highlighting this important point. We acknowledge that the predictive model for incidence rate can provide stakeholders and authorities with valuable insight to support decision-making or policy development. We have now mentioned this point as the limitation of our study (Lines: 538 – 544):

- Additionally, while our binary classification approach effectively distinguishes between high and low-risk provinces, this dichotomous categorization may mask essential gradations in risk levels. Machine learning algorithms that directly predict continuous incidence rates, rather than binary outcomes, could provide more nuanced and actionable insights for stakeholders and authorities to support decision-making.

Comment 4: When describing the problem around line 179 it may be best to be more exact (it is described exactly in the results)

Response: Thank you for your suggestion. We have rewritten the relevant sentences to improve clarity. The revised text now reads:

- For our classification model, we calculated the annual incidence rate per 100,000 population for each province as an indicator of leptospirosis outbreak level. We selected a risk classification threshold of 2.43 cases per 100,000 population, corresponding to the nationwide median incidence rate during the training period (2007-2017). This threshold effectively distinguished between provinces with elevated outbreak risk and those with baseline endemic levels, dividing the data into high- and low-risk classes.

Comment 5: Finally, although the code included covers the diagrams it would be nice if the complete code could be included i.e. hyperparameter tuning. e 49)

Response: We have revised the manuscript as suggested. The complete code, including hyperparameter tuning procedures and all additional scripts used in this study, has now been provided.

Reviewer #2

Comment 1: I am not qualified to critique application of the AI model. A key limitation of the method is the reliance on passive surveillance for identifying cases which will undoubtably under-report cases in a non-random way. In some way the authors acknowledge this with their interpretation of the 2020-2021 COVID-19 affected data. While I appreciate that a two decade active surveillance programme is not feasible, it would be good to explore this limitation.

Response: We thank the reviewer for this suggestion and appreciate their understanding of our study's limitations. To address this concern, we have added the following text to the limitations section (Lines: 530-533):

- Our data rely on a passive surveillance protocol, where under-reporting can occur non-randomly due to external factors. Reported cases from this passive surveillance system may be affected by unequal access to healthcare or limited diagnostic testing. Consequently, true incidence rates may differ from officially reported cases.

Comment 2: I was a bit concerned that when the model performed less well (post-2019) the data was excluded from analysis. Could this indicate an inability to generalise your findings?

Response: We would like to clarify that the reduced model performance during the COVID-19 pandemic is not unexpected. This decline indicates that COVID-19 impacted leptospirosis transmission through mechanisms our model could not capture. Machine learning models inherently learn patterns from their training data. In our case, the XGBoost model captured associations between leptospirosis risk and environmental and socioeconomic factors during the training period (2007-2017), a time before the unprecedented disruptions caused by COVID-19—including social distancing, remote working, and mobility restrictions. Therefore, it is expected that test data from a period with such major disruptions would yield lower predictive performance compared to normal conditions.

Despite the lower performance during the COVID-19 pandemic, we found that the SHAP importance rankings remained largely consistent when comparing the full dataset with the COVID-19 period data (Figure S5). Specifically, the top five features in the SHAP importance rankings remained unchanged, while features ranked 6th through 10th showed only minor reshuffling. This minor variation occurs because the mean absolute SHAP values of these lower-ranked features are relatively similar. The consistency in feature rankings demonstrates that our findings generalize well, as the model relies on the same key features to generate predictions regardless of the time period (Figure S5). However, the underlying patterns of leptospirosis outbreaks were fundamentally disrupted during the COVID-19 pandemic, which explains the decline in model performance during this period.

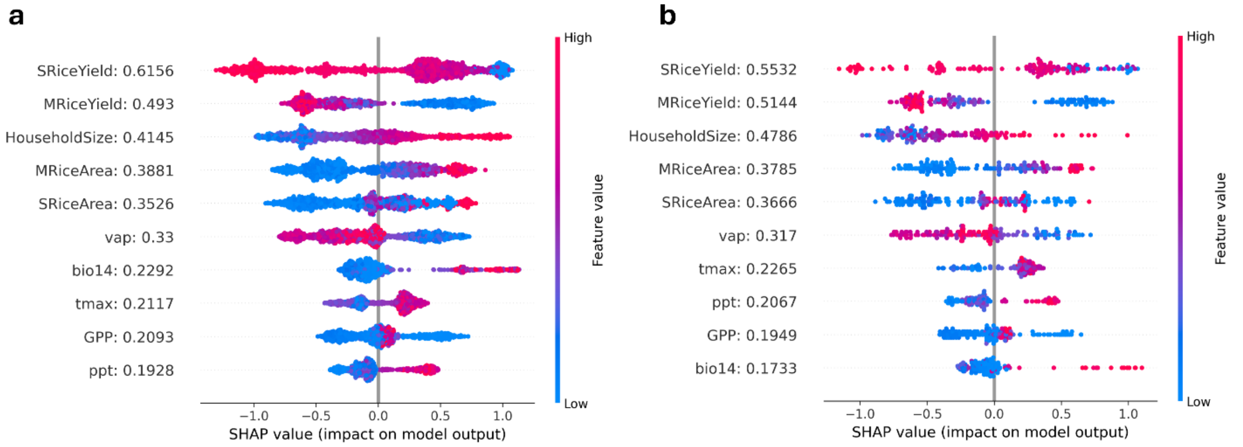

Figure S5. SHAP (SHapley Additive exPlanations) analysis identifying the top 10 predictive features for leptospirosis risk, ranked by mean absolute SHAP values. **(a)** Analysis using the complete dataset spanning 2007–2022. **(b)** Analysis restricted to the COVID-19 pandemic period (2020–2021).

Importantly, our model's performance returned to pre-COVID-19 levels in 2022, indicating that the pandemic's disruptive effects were temporary and diminished once restrictions were lifted.

To provide clarity for readers, we have revised Figure 3b in the main text to use the full dataset, thereby showing the overall feature importance rankings. An additional analysis focusing specifically on the 2020-2021 period has been provided in the supplementary file as Figure S5. Notably, the feature rankings remained consistent even when we used the full dataset instead of excluding the post-2019 data.

Furthermore, we have added the following text to the Results section (Lines: 304-309):

- Temporal stratification of model predictions revealed nuanced performance patterns. The model accurately captured leptospirosis risk dynamics prior to 2020, but systematically overpredicted risk during 2020-2021 (Fig. 3a). This overprediction pattern suggests that COVID-19-related interventions, including mobility restrictions, behavioral modifications, and altered healthcare-seeking patterns, may have suppressed leptospirosis transmission through mechanisms independent of the environmental and socioeconomic drivers incorporated in our model.

In addition, the following text has been added to Lines 351 – 356 to emphasize that our model performance recovered in 2022:

- The model's predictive performance varied significantly during the pandemic period. Annual AUC calculations revealed a notable decline in 2021, representing the lowest performance of the study period and suggesting the influence of unmeasured pandemic-related factors such as mobility restrictions, behavioral modifications, and altered healthcare-seeking patterns (Figs. 4a and S3). This pattern was consistent across all performance metrics (Fig. 4b). However, model performance recovered in 2022, returning to pre-pandemic levels with an AUC of 0.951 (95% CI: 0.861-0.999).

Also, the following text has been added to the Discussion section to discuss the impact of COVID-19 (Lines: 493 – 504):

- The deterioration in model performance during the COVID-19 pandemic is not unexpected, as our XGBoost model did not capture COVID-19-related interventions that likely disrupted leptospirosis transmission. This aligns with the typical performance decline observed in machine learning models when test set conditions

differ from the training set (1, 2). Notably, SHAP importance rankings derived from pandemic-period data remained similar to those from the full dataset (Figure S5), indicating that our model continued to rely on the same set of features to generate predictions despite the fundamentally altered epidemiological conditions during the COVID-19 pandemic. This consistency in feature importance, coupled with reduced predictive accuracy, suggests that the pandemic disrupted the established relationships between predictors and leptospirosis risk. Importantly, model performance recovered in 2022 following the lifting of pandemic control measures, achieving an AUC of 0.951 (95% CI: 0.861-0.999), demonstrating the model's robustness when applied to conditions similar to those in the training period.

Comment 3: Is it possible to place confidence intervals around your point estimates of AUC? I think these are important in considering the precision of your estimates.

Response: We thank the reviewer for this suggestion. We have now added confidence intervals for the AUC values using a bootstrapping resampling procedure (3, 4). The procedure was implemented using the "bootstrap" function in the SciPy package with 1,000 resampling iterations. Since the sci-kit learn package does not have support for native confidence intervals of AUC, the AUC plot with the 95% confidence intervals (95% CI) is obtained by interpolating the returned value of the roc_curve function to a fixed false positive rate grid to ensure a fixed number of data points across resampling iterations. Then, the confidence interval of each true positive rate was calculated using the percentile method. Finally, the lower bound and upper bound of AUC were calculated using the area under the curve acquired from the lower bound and the upper bound, respectively.

Figure 3 has been revised to include 95% confidence intervals for the AUC values. To maintain figure clarity, we have provided an additional version of Figure 4a with 95% CIs in the supplementary material as Figure S4. All AUC values reported throughout the manuscript have been updated to include these confidence interval estimates.

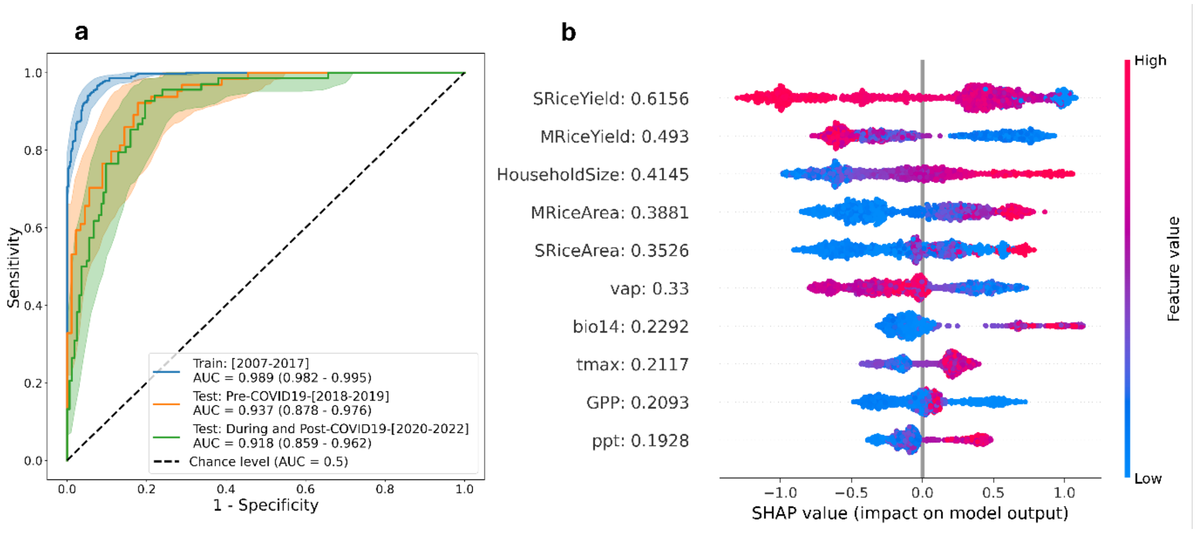

- **Figure 3.** XGBoost model performance metrics and SHAP-based feature importance analysis. **(a)** Receiver operating characteristic (ROC) curves showing model discrimination ability across different datasets, with corresponding area under the curve (AUC) values. Results are derived from the optimal hyperparameters identified through 5-fold cross-validation. The diagonal line represents random classification (AUC = 0.5), while AUC = 1.0 indicates perfect prediction. The shaded area represents 95% confidence intervals (95% CIs) based on 1,000 bootstraps (3). 95% CIs of AUC were given in parentheses. **(b)** SHAP (SHapley Additive exPlanations) analysis of the top 10 predictive features, ranked by mean absolute SHAP values (2007-2022). Individual observations are represented as dots, with color intensity indicating feature magnitude (red = high, blue = low). SHAP values on the x-axis show each feature's contribution to the predicted probability, centered at log odds = 0 (corresponding to a predicted probability of 0.5).

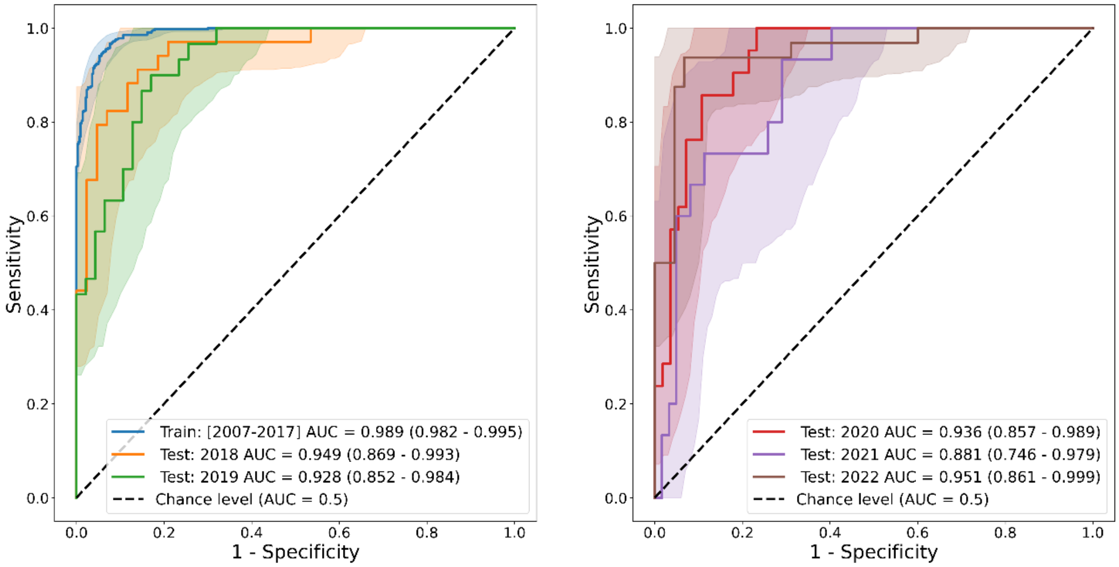

- **Figure S4**. Receiver operating characteristic (ROC) curves with area under the curve (AUC) values comparing model discrimination ability across three periods: pre-pandemic (2018-2019), pandemic (2020-2021), and post-pandemic (2022). The diagonal reference line represents random classification (AUC = 0.5), while perfect classification corresponds to AUC = 1.0. Shaded areas indicate 95% confidence intervals based on 1,000 bootstrap iterations [2]. 95% CIs of AUC are given in parentheses.

Comment 4: It would be very helpful to explore the limitations of your methods - for example, challenges with variation in reported cases might reflect variable barriers to presentation or diagnostic testing as well as variation in incidence.

Response: We thank the reviewer for identifying this important limitation. We have revised the limitations section to address this concern with the following addition (Lines: 521-533):

- First, our analysis assumes consistent case reporting outside the pandemic period, potentially underestimating actual disease burden due to unreported mild and asymptomatic cases. Our data rely on a passive surveillance protocol, where under-reporting can occur non-randomly due to external factors. Reported cases from this

passive surveillance system may be affected by unequal access to healthcare or limited diagnostic testing. Consequently, true incidence rates may differ from officially reported cases.

Comment 5: Your model reflects association rather than (neccessarily) causation, and it would be useful for readers to highlight this more.

Response: We agree with the reviewer's observation that machine learning models capture associations rather than causal relationships. To clarify this important distinction, we have added the following text to the limitations section (Lines: 542-544):

- Finally, our model and analysis identify feature associations rather than causal relationships with leptospirosis risk. More sophisticated analyses are needed to confirm causal relationships between these factors and leptospirosis risk.

We thank the reviewers once again for their valuable feedback and constructive comments, which have significantly improved the quality of our manuscript. We believe that the revised manuscript now addresses all the concerns raised, and hope that it is now suitable for publication in PLOS Neglected Tropical Diseases.

Sincerely,

Charin Modchang and Joacim Rocklöv

On behalf of all co-authors

References

1. Quiñonero-Candela J, Sugiyama M, Schwaighofer A, Lawrence ND. Dataset shift in machine learning: Mit Press; 2022.
2. Kaur H, Pannu HS, Malhi AK. A Systematic Review on Imbalanced Data Challenges in Machine Learning: Applications and Solutions. ACM Comput Surv. 2019;52(4):Article 79.
3. Carpenter J, Bithell J. Bootstrap confidence intervals: when, which, what? A practical guide for medical statisticians. Statistics in Medicine. 2000;19(9):1141–64.
4. Hesterberg T. Bootstrap. WIREs Computational Statistics. 2011;3(6):497–526.
